# How do you argue with a science denial meme? Memed responses may be counter-productive for responding to science denial online

**DOI:** 10.1177/09636625251341509

**Published:** 2025-05-31

**Authors:** Hannah Little, Justin Sulik

**Affiliations:** University of Liverpool, UK; LMU Munich, Germany

**Keywords:** analogy, argumentation, science communication, science denial, social media

## Abstract

Science denial ‘memes’ are a viral form of communication that attempt to undermine complex scientific ideas using memorable soundbites. These memes misrepresent the scientific content they are ‘debunking’, making responding to them challenging. To identify common strategies, we analysed Twitter/X responses to the anti-evolution meme ‘why are there still monkeys?’. Strategies included literal explanations about why the reasoning behind the meme is flawed, and analogies that mirror the original meme to varying degrees (e.g. in structure and/or domain). We evaluated different response strategies using an experiment with participants from the United States who either endorsed or denied evolution. Participants rated their understanding of the original meme and different response strategies, and how effective and persuasive they found them. Across participants, literal explanations were rated more understandable, effective and persuasive than analogical responses. Memed rebuttals may thus be a counter-productive strategy for responding to science denial online.

## 1. Introduction

Increasingly, science communication takes place online. With this media shift has come a proliferation of science denial and misinformation, especially on social media (Rosenau, 2012).

Much of this content takes the form of single sentence ‘memes’, a viral form of online communication attempting to undermine complex scientific ideas by pointing out seemingly obvious flaws in argumentation while making the idea more succinct and therefore more memorable and repeatable. Examples include ‘If humans evolved from monkeys, why are there still monkeys?’ and ‘If global warming is real, why is it snowing?’.

Importantly, these memes misrepresent the scientific content they attempt to debunk. This presents a double challenge for rebuttal: pointing out the fundamental misunderstanding underlying the question may sound like one is avoiding the question and thus unable to answer. Conversely, by answering the question briefly, one may be forced to gloss over the fundamental misunderstanding. So how can online communities craft concise, informative responses that constructively engage with the underlying scientific misunderstanding, while remaining suitable for social media? How do you argue with a meme?

In this paper, we present experimental data evaluating the effectiveness of various responses to such memes from the perspectives of different audiences. Focusing specifically on evolution memes, we pose the following research questions (RQs):

RQ1 How do pro-science social media users react to science-denial memes online?
RQ2 What strategies are more effective at responding to science-denial memes?

## 2. Science denial

Science denial contests claims that have broad scientific consensus supported by substantial empirical evidence. It may involve rhetorical strategies to create the appearance of legitimate argumentation without scientific objectivity (Diethelm and McKee, 2009; Oreskes and Conway, 2010). Science denial can have significant negative societal implications, for instance, when individuals become unwilling to reduce carbon emissions (Jolley and Douglas, 2014b) or do not accept vaccinations that can protect communities (Jolley and Douglas, 2014a). Science communicators therefore have a duty to try to rectify science denial beliefs.

However, since those who hold science denial beliefs are likely to distrust science and scientists (Hornsey et al., 2016; Nadelson and Hardy, 2015; Sulik et al., 2021), or may have their own (unscientific) experts and arguments (Allchin, 2021; Diethelm and McKee, 2009), it may be difficult to persuade using only scientific content. This aligns with the move, over the last few decades, away from a top- down ‘deficit view’ of the public understanding of science – focusing primarily on gaps in scientific knowledge – towards models which consider diverse audiences’ contexts and perspectives (Brossard and Lewenstein, 2009).

### Science denial online

Science denial is not a new phenomenon – it substantially predates the Internet (Jin et al., 2024). In the United States (from where we recruit our sample), science beliefs have been polarised along political and religious lines (Drummond and Fischhoff, 2017). Beyond the United States, religiosity predicts disbelief in evolution, including in countries that are not predominantly Christian (Rutjens et al., 2022), though not all religions – or, even within Christianity, all denominations – consider evolution incompatible with their beliefs (Barone et al., 2014; Martin, 2010).

There are also examples of science denial being spread by traditional mass media, such as the Wakefield scandal creating scepticism about the MMR vaccine (Cassidy, 2006) or dismissal of evidence of global warming (Elsasser and Dunlap, 2013).

However, aspects of the online environment might increase ease of connection and communication between individuals prone to science denialism, including the speed which information can be spread, the lack of editorial gatekeepers fact-checking content, and the reinforcement of beliefs by content recommendation algorithms (Sinatra and Hofer, 2021).

Motivations for denying science online can include wanting to produce clickbait or viral videos, trolling, religious fundamentalism and even politicisation of science-denial content (Paolillo, 2018). For platforms relying on user-generated content, content undermining scientific consensus may overwhelm pro-science rebuttals: flat-Earth videos on YouTube outnumber those debunking this idea (Mohammed, 2019) and on Twitter/X, misinformation relating to Covid-19 is shared more than science-based evidence or fact-checking posts (Pulido et al., 2020). There are also closed online spaces created specifically for discussion of science denialism where science-positive voices are missing, including subreddits, Discord servers, Facebook groups, WhatsApp groups, or Telegram groups.

The prevalence of science denial online does not necessarily convert audiences to denialist beliefs: some people are more or less susceptible. Factors increasing individuals’ susceptibility to science denial content include scientific knowledge, intuitive cognitive style, motivated reasoning, susceptibility to conspiracy ideation and religious or conservative worldviews (Azevedo and Jost, 2021; Gervais, 2015; Landrum et al., 2021; Lewandowsky and Oberauer, 2016; Rutjens et al., 2018a; Scherer et al., 2021; Sinatra et al., 2014). While difficult to quantify, user susceptibility remains important in evaluating strategies for effectively responding to science denial online.

### Responding to science denial

If science advocates do not respond to science denial, observers may reduce approval for science or fail to adopt behaviours consistent with science-positive attitudes (e.g. getting vaccinated, Schmid and Betsch, 2019). Therefore, it is important to evaluate effective strategies for responding to science denial. Schmid and Betsch (2019) found that factual strategies (providing facts about the science being denied) are as effective as strategies debunking rhetorical devices used by science deniers. However, their study did not provide an analysis of the form these factual responses might take. For example, factual explanations might use analogies to build on existing knowledge, or just offer straightforward explanations of the science.

Analogy is often used to explain science in both formal and informal contexts. Analogy is fundamental for human cognition (Gentner, 2010) and can be a powerful tool for science education (Duit, 1991). However, the use of analogies in science communication can sometimes lead to incorrect conclusions if people misunderstand which features or relationships within the analogy are relevant (Little, 2019). The audience for an analogy needs to have pre-existing knowledge of the analogy elements to derive the intended meaning. For example, to appreciate whether selective breeding represents a good analogy for natural evolution, one must first know how selective breeding works. Evidence from the science education literature informs what makes analogies more effective. For example, analogies with close surface similarity to the thing they are trying to explain may be more accessible (Holyoak and Koh, 1987). One way analogies can be close to the concepts they explain is when both belong to the same domain. For example, one can learn about evolution by comparing it with selective breeding: the elements in the selective breeding analogy (animals or plants) are in the same domain (biological organisms) as the evolutionary processes being explained. Analogies can also be further removed from the target concept, as when explaining aspects of biological evolution by appealing to examples from cultural evolution (e.g. the development of Roman letters from Greek ones or of modern institutions from older ones). The closer the analogy, the more likely audiences are to understand its relevance: it is easier for audiences to see the mechanistic relationship between selective breeding and natural selection than to engage in a further step of abstract conceptualisation to see why cultural evolution is relevant.

Domain closeness is not the only dimension for deriving meaning. The grammatical structure of analogies can be exploited to make the conceptual mapping between elements clearer. For example, if one was to say, ‘socks are to feet as gloves are to hands’, the mirrored syntactic structure of these two phrases makes the symmetry of the conceptual relationships (socks:feet::gloves:hands) clear. In the context of science-denial memes, a structured analogical response to ‘If humans evolved from monkeys, why are there still monkeys?’ would mirror aspects of the meme’s syntax (e.g. ‘If you’re descended from your grandparents, why do you still have cousins?’). An unstructured analogical response need not do so (e.g. ‘Same reason your birth wouldn’t cause the death of your cousins’.).

Gentner et al. (1993) argues that structural similarity between an analogy and its subject enables more complete and accurate learning. Without structural similarity, it may be harder to interpret why someone is saying that gloves are like socks: it could be due to their size, colour, or material, rather than their use.

Responses to science denial might also differ in the accessibility of the language used, the framing of the explanation, the sources cited, and the politeness of the language. Even an accurate explanation can still be unsatisfying from an audience’s perspective. For example, Sulik et al. (2023) showed that laypeople rated explanations as more satisfying when they included information on mechanism (how something works) and function (its goal or purpose). They found that people were generally good at assessing whether an explanation was accurate, but were less good at judging how satisfying explanations might appear to others. Therefore, it is crucial to bear different audiences (e.g. science-affirming versus science-denying) in mind when developing strategies for combating science denial.

Given this literature, we want to evaluate the effectiveness of different structures that factual responses can take, both from the perspective of people who believe the science being debunked, and those who do not. We are specifically interested in how analogical responses compare to literal ones. The literature on analogy also motivates us to assess whether analogical responses are more effective with close surface similarity to the meme, with structural similarity to the meme, or when taking advantage of pre-existing knowledge that evolution sceptics are likely to possess.

## 3. Study overview

The current study seeks to establish effective structures that responses to online science denial memes online can take. To fulfil this objective, we first identify what strategies people are using to respond to science denial on Twitter/X^1^; second, we recruit an online sample of people to rate the effectiveness of these responses.

The study focuses on one science denial meme: ‘If humans descended from monkeys, why are there still monkeys?’ or ‘If apes descended from monkeys, why are there still apes?’. We chose this as it captures many of the core features of memes and the challenges science communicators face in responding to them. It is succinct, memorable, repeated often in online spaces, and misrepresents the science it attempts to debunk.

We are mindful that this meme has religious connotations, because evolution denial is often the result of creationist beliefs, which are particularly prominent in certain Christian denominations (Drummond and Fischhoff, 2017; Martin, 2010). This religious dimension may impact responses in a way that differs from memes that lack this dimension. Despite this potential limitation on generality, it also allows us to consider how religion might contribute alongside other factors, such as (dis)trust in science, to how people respond to such memes.

To establish what strategies and structures people are using to respond to science denial, we first built a corpus of responses to the evolution meme on Twitter/X and conducted a qualitative analysis of these responses, which then informs the main study of the paper. The main study uses an online rating task to assess the success of different response strategies using two groups of participants: evolution endorsers and evolution sceptics.

## 4. Pre-study: Social media corpus creation and analysis

### Methods

To establish what strategies people currently use to respond to our meme, we built a database of responses to the monkey meme from Twitter/X. Using Twitter’s search function, we found a number of versions of this meme involve the question ‘why are there still monkeys?’ or ‘why are there still apes?’. We stored the first 100 responses found with a science communication agenda, loosely construed as responses that were trying to counter the meme, either by arguing against its content or discrediting the poster or idea. Responses agreeing with the reasoning of the meme or asserting that evolution was false were omitted. We also omitted racist responses, even if racism was being used as a way to discredit the original poster, as racism is not consistent with a science-positive agenda.

A more systematic approach in scraping this data with Application Programming Interfaces (APIs) could have captured a higher number of responses. However, the purpose of this pre-study was to identify items to be used in our main rating study, so a more light-touch approach seemed sufficient.

Coding was done by one author. Thematic analysis of responses within the database was achieved in two steps. First, tweets were inductively coded into themes by categorising the strategies used in responding to the meme. A second, more fine-grained, inductive thematic analysis coded for common subject domains and themes used in responses. All coding was conducted using the iterative steps from Braun and Clarke (2012).

### Results

Response strategies fitted into three main categories: the majority involved language intended to offend or belittle the original poster (42%), followed by responses explaining some factual element of evolution (32%). Responses using analogy to explain some evolutionary process made up 28% (percentages don’t sum to 100 because some responses combined response types). All response types were well-represented in our sample.

Most tweets aimed at belittling or offending the original poster made reference to the poster being stupid and lacking knowledge (63%). 17% made reference to the original tweet by comparing the poster with being a monkey or ape. 24% referenced education levels, some specifically mentioning school-level education or criticising the poster for not self-educating. 7% mentioned scientists within their insults, as figures who would oppose the meme’s content.

For straightforward factual responses, these were exclusively in the domain of science and evolution, using concepts such as phylogenetics, genetics, common ancestors and survival.

For analogical responses, 29% were in the domain of religion (e.g. ‘If man was made from mud, why is there still mud?’ and ‘If the Church of England originated by splitting off from the Catholics, why are there still Catholics?’).

Another 25% appealed to the domain of ancestry (e.g. ‘If you are descended from your grandparents, why do you still have cousins?’). These are close surface analogies, using the same referent for one aspect of the analogy (humans), explaining why the original meme is logically flawed by pointing to immediate familial relationships that audiences should be familiar with.

25% of analogies were biological. These, too, are close surface analogies, but instead of using familial relationships, they tended to rely on knowledge of selective breeding to illustrate the flawed logic. For example, ‘If dogs evolved from wolves, why are there still wolves?’.

Other analogies were drawn from domains further from evolution and biology. Technology was the most popular among these other domains, including examples of cultural evolution and technological progression. For example, ‘If we have space shuttles, why are there still cars?’ or ‘How do bicycles still exist when we have motorbikes?’.

Nearly all analogies were structural analogies, using the same syntactic and conceptual structure as the original post. Most tried to highlight why a linear understanding of evolution is flawed, pointing to analogues that obviously branch from each other. These responses used examples of biological or cultural descendants with a common ancestor, such as cousins. However, some analogies pointed to non-branching processes. For example, ‘If butterflies evolved from caterpillars then why are there still caterpillars’ points to a developmental rather than evolutionary process.

## 5. Main study: Online rating experiment

### Methods

#### Participants

To evaluate the response strategies identified during the social media analysis (pre-study), we used CloudResearch’s MTurk Toolkit (Litman et al., 2017) to recruit participants living in the United States for online rating tasks. Participants were recruited in two stages.

In Stage 1, we recruited participants (N = 1413) to complete a set of multiple-choice English proficiency questions and multiple-choice questions about their science literacy drawn from Allum et al. (2018); Kahan (2017); Miller (1998); Rutjens et al. (2018b); Sherkat (2011). For items, see Appendix 1 in the Supplemental Material. An instructional manipulation check was included among the science literacy items (‘Which of these is the densest? Please select ‘air’’. Response options were ‘water’, ‘ice’, ‘air’ and ‘don’t know’).

The English proficiency items and the instructional manipulation check provide an extra layer of data quality assurance, beyond that offered by CloudResearch, in keeping with best practice for online research (Cuskley and Sulik, 2024). To be eligible for re-recruitment in Stage 2, participants had to follow the instruction to select ‘air’ for the attention check (1273 passed), and had to score at least 70% on the English proficiency question (1358 passed). A total of 1121 passed both criteria and were thus eligible for re-recruitment. Figure S1 in the Supplemental Material shows how data-quality criteria were distributed across the science literacy scale.

In Stage 2, we aimed to re-recruit 500 participants from Stage 1, split evenly between those who had responded ‘true’ to the statement that ‘human beings developed from earlier species of animals’ (one of the science literacy questions) and those who did not agree with this item (either by responding ‘false’ or ‘don’t know’, either way failing to endorse the scientific consensus).

We ultimately recruited n = 505 (of whom 250 endorsed evolution; 255 who did not). As 1004 had endorsed evolution in the Stage 1 sample whereas 409 had not, our Stage 2 sample is purposefully not representative: evolution deniers would otherwise be represented by a small sample size in Stage 2, increasing uncertainty around model parameters or obscuring between-group differences.

#### Materials

In Stage 2, each participant evaluated four items across four main conditions: the original meme (‘If people are descended from monkeys, then why are there still monkeys?’) and three different pro-evolution rebuttals. For all participants, these included one literal rebuttal and two kinds of analogical rebuttal. Thus, these main conditions are fully within-subject.

Literal rebuttals explicitly pointed out errors in the monkey meme, paraphrased from rebuttals from in the database of responses found on Twitter/X (Pre-study). Each participant was randomly assigned one of the following literal rebuttal item variants:

1. This question assumes we are descended from monkeys, but actually monkeys are more like our cousins than our ancestors.
2. This question assumes that evolution works in a straight line, but actually it’s more like a tree, so one species appearing doesn’t mean that another species has died out.
3. This question assumes that humans would have to take the place of an earlier species, but new species often arise because they moved to a different place from their ancestors.

Each participant was also assigned two analogical rebuttals. One was a structural analogy mirroring the syntax of the original meme (e.g. ‘If you are descended from your grandparents, why do you still have cousins?’). The other was a non-structural analogy that departed from the meme syntax (e.g. ‘Same reason your birth wouldn’t cause the death of your cousins’.). For other analogical rebuttal variants, see Table 1.

**Table 1.** Analogical rebuttals used in the rating study. For each combination of domain and structure, there were two item variants.

Domain	Structure	Item variants
Religion	Unstructured	Same reason God creating people from dirt didn’t stop there being any dirt.
		Same reason Protestants splitting off from Catholics didn’t stop there being any Catholics.
	Structured	If God created us from dirt, why is there still dirt? Same logic.
		If Protestants originated by splitting off from the Catholics, why are there still Catholics? Same logic.
Ancestry	Unstructured	Same reason your birth wouldn’t cause the death of your cousins.
		Same reason that an American having Irish ancestry doesn’t mean there are no more people in Ireland.
	Structured	If you are descended from your grandparents, why do you still have cousins? Same logic.
		If some American has ancestors from Ireland, why are there still people in Ireland? Same logic.
	Unstructured	Same reason the invention of motorbikes doesn’t stop there being any bicycles.
		Same reason the invention of cell phones doesn’t stop there being any landlines.
	Structured	If we have motorbikes, why are there still bicycles? Same logic.
		If we have cell phones, why are there still landlines? Same logic.

As an additional partly within- and partly between-subject factor, analogies were drawn from three potential domains: ancestry (as in the previous examples of grandparents and cousins), religion (e.g. involving splits between denominations), and technology (e.g. comparing motorbikes and bicycles). Each participant was randomly assigned two out of these three analogy domains. One of the two was randomly selected to be structured, the other unstructured, as per the main experimental conditions.

For each of the four items, participants made three evaluations, using a percentage slider scale to assess (1) understanding, (2) effectiveness and (3) persuasion:

1. Can you see what point this *[response*^2^*]* is trying to make (regardless whether you agree with it or not)?
2. How effective do you think the reasoning is?
3. How persuaded are you by this *[response]*?

Separating these three outcome measures was important, as understanding an argument is distinct from being persuaded by it, which in turn is different from recognising that, even if it does not persuade oneself, it may be effective in general.

The monkey meme was always presented first. After rating the monkey meme, participants were also asked to explain in their own words what point they thought it was trying to convey (qualitative data not analysed here). Three rebuttals followed in a randomised order. Finally, participants answered questionnaires about their (dis)trust in science (Hartman et al., 2017, reliability *ω_h_* = 0.91, *α* = 0.95) and religious beliefs (Jong and Halberstadt, 2016, *ω_h_* = 0.95, *α* = 0.97), as well as basic demographics (age, gender, level of education).

### Analysis strategy

In analysing our outcome variables using regression models, we note that several features make linear (Gaussian) regression unsuitable. Responses were on a percentage scale (handled as a proportion here with ‘100%’ mapped onto 1). Thus, the variables are bounded between 0 and 1. Furthermore, many responses lay on those boundaries (i.e. responses frequently clustered at the extremes, at 0 and at 1, as illustrated in Figure S2). Although linear regression can work practically in some cases even when it is theoretically unsuitable, these features make it particularly problematic here.

Accordingly, we modelled outcomes with generalised Bayesian regressions. We specified zero-one-inflated Beta regressions (using R package brms Bürkner, 2021), as these are a sensible way to handle spikes at the extremes of a scale that is bounded between 0 and 1. These models include three components, namely, (1) a logistic ‘zero-one inflation’ (ZOI) component to model the probability that a participant chose an extreme response (responding with *either* 0 *or* 1); (2) a logistic ‘conditional one inflation’ (COI) component to model the probability that they chose a 1 *rather than* 0, conditional on being extreme in component 1; (3) a Beta component to model the mean and dispersion of responses that lie between 0 and 1 (conditional on not being extreme in component 1).

We apply this family of models to understand how item ratings vary as a function of

1. Outcome **scale** (values ‘understand’, ‘effective’ or ‘persuasive’);
2. Participants’ **evolution endorsement** (‘yes’ meaning that participants selected ‘true’ in response to the statement that humans developed from earlier species of animals; ‘no’ meaning that participants failed to endorse this scientific consensus, either by selecting ‘false’ or ‘don’t know’);
3. Item **stance** (values ‘monkey meme’ or ‘rebuttal’);
4. Rebuttal **type** (‘literal rebuttal’ or ‘analogical rebuttal’);
5. Analogy **structure** (values ‘structured’ or ‘unstructured’);
6. Analogy **domain** (values ‘ancestry’, ‘religion’ and ‘technology’).

The results proceed from coarse-grained to fine-grained, first just comparing the anti-evolution monkey meme with all rebuttals taken together, then adding further detail by contrasting literal and analogical rebuttals, then structured and unstructured analogies, then different analogy domains. Finally, we consider responses in the broader context of attitudes to science and religious beliefs, to ensure that our results do not depend too strongly on one specifically worded item about evolution.

All models include a by-participant random intercept and random slopes for the main predictors (except for evolution endorsement, because each participant only has one value here). We chose a weakly informative prior for model fixed effects in the Beta component (*b* ∼ *N* (0, 1)). To aid convergence, we placed slightly stronger priors on the fixed effects in the logistic components and on predictors of the precision parameter (both *N* (0, 0.5)).

We report conditional effects or average comparisons between conditions using (R package marginaleffects Arel-Bundock, 2024). This incorporates the model components mentioned above (the Beta and logistic components) while adding together interaction terms to reach model-based predictions for specific conditions or combinations of predictors.

Regression outputs and marginal effects are in Tables S2 to S9, while the full analysis script is available at https://osf.io/kfzax/.

### Results

#### Descriptive overview

While our main aim is to evaluate different communication strategies for rebutting the monkey meme, it is worth first understanding potential audiences for those strategies. We therefore provide a brief overview of how participants who did not endorse evolution – either by responding ‘false’ (n = 136) or ‘don’t know’ (n = 119) to the evolution item in the science literacy scale – differed from those who did.

Summary statistics for demographic and psychological variables are in Table 2 and Figure S3. Overall, those who did not endorse evolution were older (*b* = 4.14, *t* = 3.98, *p* *<* .001), more likely to be women (χ^2^ = 25.95, *df* = 2, *p* *<* .001), more religious (*b* = 1.76, *t* = 14.6, *p* *<* .001), more distrustful of science (*b* = 1.08, *t* = 10.43, *p* *<* .001) and had lower science literacy (*b* = −0.22, *t* = −13.18, *p* *<* .001). However, they did not differ significantly in reported education level (*b* = −0.077, *t* = −1.24, *p* = 0.216).

**Table 2.** Descriptive statistics for demographic and psychological variables (Stage 2 recruitment). Those who do not endorse evolution include participants who responded ‘false’ to the evolution item (n = 136) and those who responded ‘don’t know’ (n = 119).

		Endorse evolution?	
	Total	Yes	No
	(N = 505)	(N = 250)	(N = 255)
**Age**			
Mean (SD)	41.2 (11.9)	39.1 (11.4)	43.3 (12.0)
Median [Min, Max]	39.0 [20.0, 78.0]	37.0 [20.0, 71.0]	43.0 [22.0, 78.0]
**Gender**			
Man	261 (51.7%)	155 (62.0%)	106 (41.6%)
Woman	238 (47.1%)	90 (36.0%)	148 (58.0%)
Non-binary	6 (1.2%)	5 (2.0%)	1 (0.4%)
**Education level**			
Mean (SD)	2.87 (0.694)	2.90 (0.682)	2.83 (0.705)
Median [Min, Max]	3.00 [1.00, 5.00]	3.00 [2.00, 5.00]	3.00 [1.00, 5.00]
**Religious belief**			
Mean (SD)	3.00 (1.61)	2.12 (1.57)	3.88 (1.10)
Median [Min, Max]	3.17 [0, 5.00]	2.00 [0, 5.00]	4.17 [0, 5.00]
**Distrust in Science**			
Mean (SD)	1.97 (1.28)	1.42 (1.11)	2.50 (1.22)
Median [Min, Max]	2.00 [0, 5.00]	1.33 [0, 4.83]	2.50 [0, 5.00]
**Science literacy**			
Mean (SD)	0.668 (0.214)	0.777 (0.174)	0.561 (0.194)
Median [Min, Max]	0.710 [0, 1.00]	0.790 [0.140, 1.00]	0.570 [0, 1.00]

To contextualise beliefs about evolution relative to unscientific beliefs more generally, Table S1 shows the Spearman rank correlations between response accuracy for the evolution item and accuracy for all other items. Failure to endorse evolution is moderately associated with rejecting the Big Bang (item 7, *ρ* = 0.481), the safety of GMO foods (item 13 *ρ* = 0.325), continental drift (item 8 *ρ* = 0.299) and anthropogenic climate change (item 12 *ρ* = 0.272).

#### Regression models

**Figure 1.**
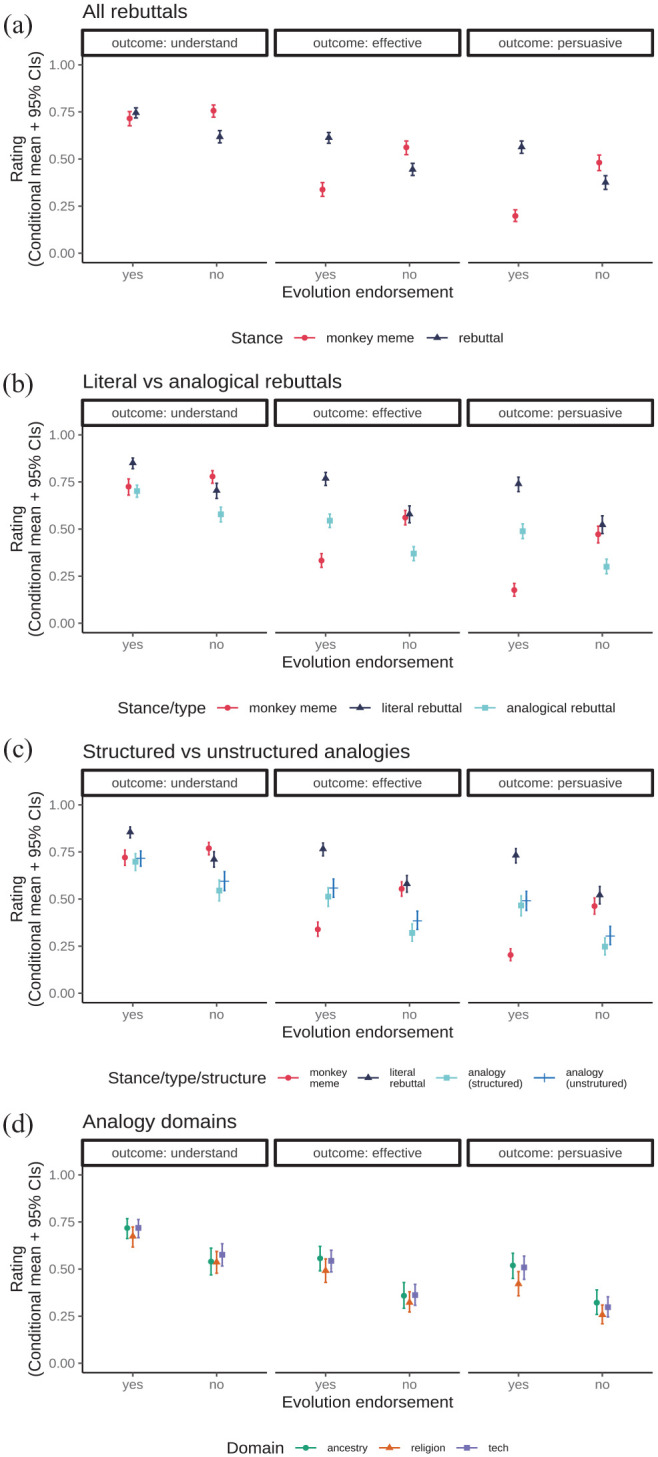
Expected values of the model posterior predictive distributions (conditional means and 95% Credibility Intervals) for item ratings as (a) a function of rating scale, item stance and participant evolution endorsement; (b) the addition of item type; (c) the addition of analogy structure; (d) analogy domain.

Figure 1 plots expected values of the models’ posterior predictive distributions (conditional means with 95% Credibility Intervals/CIs) for rated outcomes as a function of (a) participant evolution endorsement and item stance; (b) evolution endorsement, item stance and rebuttal type; and (c) evolution endorsement, item stance, item type and analogy structure.

Contrasting the monkey meme with all rebuttals (Figure 1a, Tables S2 and S3), participants who endorsed evolution were unbiased in that they reported similar levels of understanding for both the monkey meme and rebuttals (understanding of monkey meme *M* = 0.715 [0.676, 0.752]; understanding of rebuttals *M* = 0.745 [0.718, 0.772]; average comparison rebuttals – monkey meme = 0.030 [−0.015, 0.076], where the CIs include 0).

In contrast, participants not endorsing evolution showed some own-side bias, reporting understanding the monkey meme better than the pro-evolution rebuttal items (understanding of monkey meme *M* = 0.757 [0.722, 0.787]; understanding of rebuttals *M* = 0.618 [0.586, 0.651], average comparison rebuttals – monkey meme = −0.138 [−0.181, −0.091]).

Apart from rated understanding, participants who endorsed evolution found the rebuttals more effective and persuasive on average than the monkey meme. The reverse pattern was found for participants who did not endorse evolution, whereby the monkey meme was rated more effective and persuasive (see Table S3).

Contrasting literal and analogical rebuttals (Figure 1b, Tables S4 and S5), literal rebuttals were rated higher than the analogical ones overall (average comparison literal – analogical = 0.197 [0.164, 0.228]). This held for all outcome scales, whether participants endorsed evolution or not (see Table S5). Notably, while evolution deniers claimed to understand the literal rebuttal worse than the monkey meme, they rated the literal rebuttal and monkey meme as similarly effective and persuasive (see Table S5).

Expanding further by contrasting structured and unstructured analogies (Figure 1c, Tables S6 and S7), structured analogies were rated worse than unstructured ones overall (average comparison structured – unstructured = −0.044 [−0.080, −0.007]). The effects of analogy structure were generally small compared to the effects of outcome scale, item stance or rebuttal type (see Figure 1c). Thus, when broken down by outcome scale and evolution endorsement (for which, see Table S7), although the direction of the difference was consistently negative, the CIs for many conditions included 0.

Next, we consider whether the domain of the analogical rebuttals affected ratings. Due to a software bug, our data did not record the assigned domains for 19 participants (10 who endorsed evolution; 9 who did not).

As with analogical structure, the effects of analogy domain were relatively small (Figure 1d, Tables S8 and S9). Overall, religion-based analogies had worse ratings (average comparisons: religion–tech = −0.050 [−0.097, −0.005]; religion–ancestry = −0.052 [−0.102, −0.003]). Ancestry and tech domains were rated similarly (average comparison tech–ancestry = −0.001 [−0.053, 0.050], where CIs include 0). This pattern is consistent in its direction across participant evolution endorsement, not driven by evolution-deniers being differently sensitive to religious topics (see Table S9; for most conditions, CIs now include 0).

We also consider other psychological factors associated with evolution endorsement: distrust in science, science literacy and religious belief (cf. Table 2). The above results are not solely a matter of people’s specific beliefs about evolution, as they can also be considered in the broader context of worldviews and world knowledge. Accordingly, in the remaining analyses below we replace evolution endorsement as a predictor with each of these psychological variables in turn.

We caution that our sampling strategy – re-recruiting participants based on whether they endorsed a specific evolution literacy item – means that the following effects should not be assumed to generalise beyond this sample: they merely contextualise the main findings above. We focus here at the level of item type (i.e. the same granularity as in Figure 1b). We also note, too, that in the following, we model each outcome scale in a separate regression, due to issues of convergence when attempting to handle everything via interactions in a single model.

**Figure 2.**
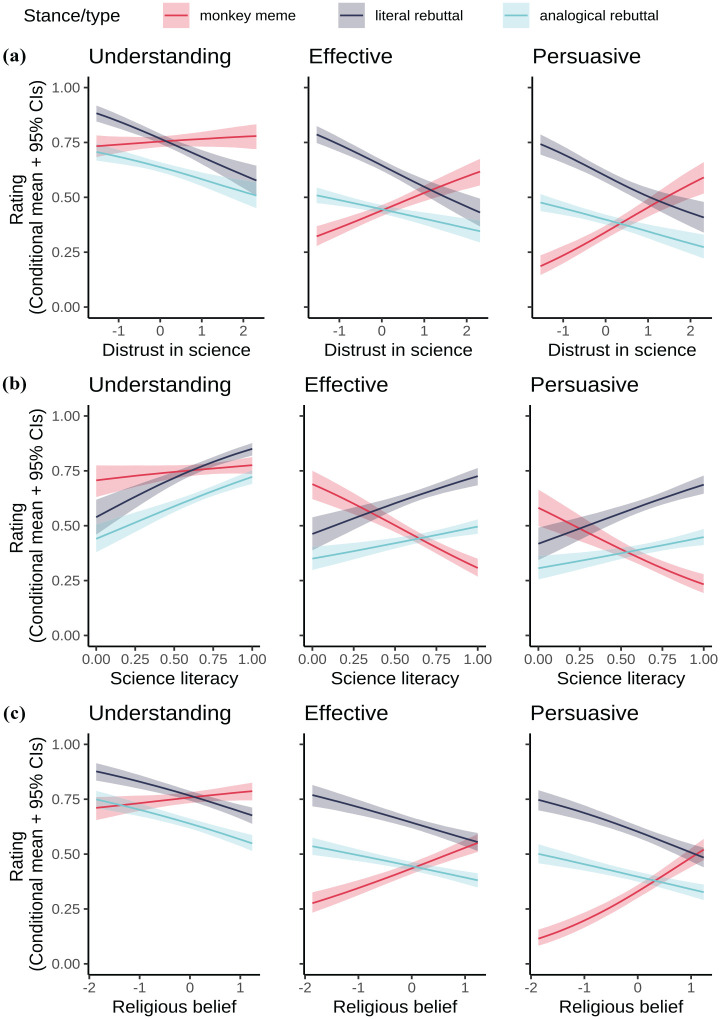
Expected values of the model posterior predictive distributions (conditional means and 95% credibility intervals) for rated understanding, effectiveness and persuasion, each in separate regressions as a function of item type/stance and (a) distrust in science; (b) science literacy; (c) religious belief. Note that higher science literacy is associated with lower evolution endorsement, so conceptually, sub-figure (b) is the mirror image of the other sub-figures here and of Figure 1. Please see online version for colour figure.

Distrust in science (Figure 2a) did not matter much for reported understanding of the monkey meme, in the sense that the coefficient was near zero and the CIs included 0 (average slope *b* = 0.006 [−0.016, 0.028]). Distrust was, however, associated with higher ratings of the monkey meme on other outcomes (effective *b* = 0.081 [0.056, 0.104]; persuasive *b* = 0.101 [0.076, 0.126]). Higher distrust was associated with lower ratings of all rebuttals on all outcomes (understanding literal *b* = −0.075 [−0.094, −0.055]; understanding analogical *b* = −0.058 [−0.076, −0.039]; effective literal *b* = −0.096 [−0.118, −0.075]; effective analogical *b* = −0.045 [−0.065, −0.026]; persuasive literal *b* = −0.098 [−0.121, −0.073], persuasive analogical *b* = −0.050 [−0.070, −0.029]).

Science literacy (Figure 2b) yields a mirror image of these patterns, as being broadly pro-science involves *higher* science literacy but *lower* distrust. The association between science literacy and reported understanding of the monkey meme has CIs that include 0 (average slope *b* = 0.077 [−0.004, 0.156]), but increased literacy was associated with lower ratings for the monkey meme on the other outcomes (effective *b* = −0.357 [−0.448, −0.261], persuasive *b* = −0.295 [−0.393, −0.196]). Higher literacy was associated with higher ratings of all rebuttals on all outcomes (understanding literal *b* = 0.257 [0.184, 0.328]; understanding analogical *b* = 0.244 [0.173, 0.316]; effective literal *b* = 0.251 [0.157, 0.341]; effective analogical *b* = 0.140 [0.067, 0.216]; persuasive literal *b* = 0.264 [0.167, 0.364]; persuasive analogical *b* = 0.133 [0.054, 0.209]).

Much the same was observed for religious beliefs: the same pattern as for distrust in science and the mirror image of science literacy. Religious belief did not matter much for reported understanding of the monkey meme (average slope *b* = 0.0127 [−0.009, 0.036]) but was associated with higher ratings of the monkey meme on other outcomes (effective *b* = 0.092 [0.067, 0.115]; persuasive *b* = 0.132 [0.106, 0.156]). Higher religious belief was associated with lower ratings of all rebuttals on all outcomes (understanding literal *b* = −0.066 [−0.088, −0.044]; understanding analogical *b* = −0.067 [−0.087, −0.047]; effective literal *b* = −0.075 [−0.098, −0.050]; effective analogical *b* = −0.051 [−0.070, −0.031]; persuasive literal *b* = −0.092 [−0.116, −0.067]; persuasive analogical *b* = −0.052 [−0.073, −0.033]).

Thus, the models with these other psychological variables broadly recapitulate the main results based on evolution endorsement: these patterns are likely not due to the specific wording of one science literacy item about evolution.

Finally, Figure S4 provides a more nuanced view by showing how participants who responded ‘false’ and ‘don’t know’ to the evolution item (both of which count as failing to endorse evolution in all previous analyses) were similar in that they rated analogical rebuttals worse than either the literal rebuttals or original monkey meme across outcomes. In other respects, the ‘don’t know’ responses are intermediate between those who said evolution is true and those that said it is false.

## 6. Discussion

This study first explored how people respond to science denial memes online, and second, evaluated various communication strategies for responding to memes, from the perspectives of diverse groups.

Our pre-study analysis suggested a strong appetite for engaging with and countering science denial memes online. While responses were often antagonistic, the majority attempted to engage with the substance of the monkey meme or to correct its central misconception. Where tweets were antagonistic, insults focused on a lack of knowledge or education, while our rating study ironically showed no association between level of education and belief in evolution (though there was an association with science literacy).

Responses focusing on correcting the misinformation of the science-denial meme unpacked why the logic of the meme was mistaken, either through a straightforward factual explanation, or through an analogy highlighting the flawed assumptions of the original tweet. These analogies used various domains, including biology, religion and technology. Analogies mostly used biology or ancestry, suggesting that those responding intuited that close surface analogies would be more effective, something also suggested by previous literature. However, the results from the rating study found that tech-based analogies were consistently rated similarly to ancestry-based ones.

After biology and ancestry, religion was the most common analogical domain in our pre-study, perhaps because evolution denial is associated with alternative accounts for creation from specific religious groups. Plausibly, pro-evolution social media users were – encouragingly – trying to take their audience’s perspective. However, religious analogies were consistently rated lower than other domains, suggesting this strategy is unlikely to achieve the intended effect.

Evolution deniers were more likely to be older, women, and to have lower scientific literacy and higher distrust of science. Some items in our test of scientific literacy were more strongly associated with evolution denialism than others, especially denial of the Big Bang. This may be because the Big Bang theory is inconsistent with interpretations of creation stories from some religions (particularly in the US context; Drummond and Fischhoff, 2017), which might also be associated with evolution denial. Rejection of continental drift was also moderately correlated with evolution denial, which again may be due to beliefs about the age of the Earth from Young Earth Creationists. Moderate correlations were also found for rejection of genetically modified foods, vaccines, and anthropogenic climate change. While these items relate less to religious belief, their social context in the United States includes mutually reinforcing associations with political ideology, conspiracy theories and distrust in science (Lewandowsky et al., 2013; Nadelson and Hardy, 2015).

Our questions aimed to establish differences between understanding pro- or anti-science messages and finding them effective and persuasive. We observed distinct patterns of responses to these different aspects of communication. These results suggest the importance of going beyond mere comprehension when studying the effectiveness of science communication in social media contexts. Many of these distinctions were as expected: evolutionists found rebuttals more effective and persuasive than the original meme, while the reverse was true for those who did not endorse evolution. Evolutionists reported understanding both the original meme and rebuttals of the meme to a similar degree, indicating that their understanding was not overtly biased by their own beliefs. However, participants who did not endorse evolution indicated that they understood the monkey meme better than the rebuttals. It is not clear whether this reflects genuine non-comprehension or else expressive responding, whereby stated beliefs reflect social identities rather than genuine epistemic commitments (Ross and Levy, 2023; Schaffner and Luks, 2018).

All groups found literal rebuttals of the meme more comprehensible, effective and persuasive than analogical responses, running counter to our expectations. Evolution deniers even rated the literal rebuttals as effective and persuasive as the original meme. However, variability in distrust in science and science literacy likely plays a role here, given how ratings of the monkey meme and the literal rebuttals fully cross over (rather than just overlapping) at the non-scientific extremes of the x-axes in Figure 2a,b (unlike in Figure 1b or Figure 2c, where they do overlap for effectiveness and persuasion). Consistent with previous literature highlighting these factors (Nadelson and Hardy, 2015), they are worth considering in future work evaluating communication strategies countering anti-science memes.

Our literature review suggested that structured analogies should be clearer than unstructured ones. However, our results showed that unstructured analogies were rated better than structured analogies. Surprisingly, then, attempting to memeify an analogy by matching the syntactic structure of a science denial meme may fail as a communication strategy. Future work might explore whether this holds for other non-syntactic forms of memeificiation or examine reasons for the observed pattern: anti-evolutionists could find memeified rebuttals disparaging, but our data do not speak to this issue.

## 7. Limitations

This paper’s primary limitation is that it only analyses one meme. However, it provides a set of rich data outlining how people respond to online science denial, how different people perceive those responses, and how perceptions interact with attitudes towards science.

Measuring attitudes or beliefs associated with evolution is hotly debated. In our study, only one science literacy item pertained to evolution, but more nuanced validated instruments exist (Nadelson and Hardy, 2015; Romine et al., 2018; Short and Hawley, 2012; Smith et al., 2016) which may shed further light. Collection of further data on the religious beliefs and denominations of participants may also provide a more nuanced analysis.

Our study is also limited to strategies found in data extracted from Twitter/X. Perhaps other theoretically-motivated strategies exist to respond to science denial that are more effective than those represented in our data. In particular, as science communication takes many diverse forms beyond online responses to memeified tweets, further work would be needed to identify and test these strategies.

Our study also does not address what makes online posts go ‘viral’. Weng et al. (2012) found that the intrinsic value of memes, such as their content or appeal, was not sufficient to explain their virality. While our rating study involved online participation, the experiment itself was not embedded in social media. Not all online environments are alike, including variability in the extent to which accuracy or truth motivates people’s decision making when encountering misinformation (Pennycook et al., 2020). Further work could investigate how extrinsic factors, such as social network structures and realistic social media environments, might contribute to both the proliferation of science-denial memes and the communicative success of pro-science responses.

Finally, our sample was recruited from the United States, where trust in science is more ideologically polarised than in many other countries, including in media and going beyond evolution (Czarnek et al., 2021; Diehl et al., 2021; Pechar et al., 2018; Sulik et al., 2021). Potential cross-cultural variation in these issues is thus a further limitation, and an important avenue for further research.

## 8. Conclusion

Our results show clear differences between how evolutionists and evolution deniers understand and react to science-denial memes and responses to them.

Awareness of these differences was evident in responses to the science-denial meme. For instance, given the prominent role of creationist beliefs in anti-evolution attitudes, responders often chose analogical examples from religion to highlight the scientific misunderstanding. However, people in our rating experiment found analogies in this domain less persuasive.

While many responders attempted to memeify their posts, mirroring the original meme in its structure, our study showed this tactic to be less comprehensible, effective and persuasive. Evolution deniers found straightforward, factual responses just as effective and persuasive as the original monkey meme, suggesting this might be the most effective strategy for countering pithy anti-science messages online.

## Supplemental Material

sj-docx-1-pus-10.1177_09636625251341509 – Supplemental material for How do you argue with a science denial meme? Memed responses may be counter-productive for responding to science denial online Supplemental material, sj-docx-1-pus-10.1177_09636625251341509 for How do you argue with a science denial meme? Memed responses may be counter-productive for responding to science denial online by Hannah Little and Justin Sulik in Public Understanding of Science

## Notes

1. At the time of data collection, it was still called Twitter. Though it is now known as X, we refer to it as ‘Twitter/X’ throughout this paper.

2. The phrasing used ‘question’ in cases where the item was a (rhetorical) question, as in the original meme, and ‘response’ where the item was a sentence.
