# Supplementary material for: How do you argue with a science denial meme? Memed responses may be counter-productive for responding to science denial online
Source: Public Underst Sci. 2025 May 31;34(8):1088–106. doi: 10.1177/09636625251341509 (PMC12535622; doi:10.1177/09636625251341509)
Supplement: sj-docx-1-pus-10.1177_09636625251341509 – Supplemental material for How do you argue with a science denial meme? Memed responses may be counter-productive for responding to science denial online [file sj-docx-1-pus-10.1177_09636625251341509.docx]

**How do you argue with a science denial meme?**

**Meme-ing responses may be counter-productive for responding to science denial online**

**Supplemental Materials**

**Hannah Little^1^ and Justin Sulik^2^**

**^1^** Department of Communication and Media, University of Liverpool

**^2^** Cognition, Values & Behavior Lab, Munich Interactive Intelligence Initiative, LMU Munich

**Contents**

1. Appendix 1: Questionnaire items
2. Figure S1
3. Figure S2
4. Figure S3
5. Table S1
6. Table S2
7. Table S3
8. Table S4
9. Table S5
10. Table S6
11. Table S7
12. Table S8
13. Table S9
14. Figure S4

**Appendix 1: Questionnaire items**

*Stage 1 recruitment*

15 science literacy items were drawn from [Miller](#_bookmark50) ([1998](#_bookmark50)); [Kahan](#_bookmark37) ([2017](#_bookmark37)); [Allum et al.](#_bookmark22) ([2018](#_bookmark22)); [Sherkat](#_bookmark48) ([2011](#_bookmark48)); [Rutjens](#_bookmark39) [et al.](#_bookmark39) ([2018b](#_bookmark39)). Response options follow, with the correct response in bold. Item 15 is the evolution item used to group participants for re-recruitment in Stage 2. The center of the earth is very hot. [**True**, False, Don’t know]

1. All radioactivity is man-made. [True, **False**, Don’t know]
2. It is the father’s genes that decide whether the baby is a boy or a girl. [**True**, False, Don’t know]
3. Lasers work by focusing sound waves. [True, **False**, Don’t know]
4. Electrons are smaller than atoms. [**True**, False, Don’t know]
5. Antibiotics kill viruses as well as bacteria. [True,

**False**, Don’t know]

1. The universe began with a huge explosion. [**True**, False, Don’t know]
2. The continents on which we live have been moving their locations for millions of years and will continue to move in the future. [**True**, False, Don’t know]
3. Does the Earth go around the sun, or does the sun go around the Earth? [**Earth around sun**, Sun around earth, Don’t know]
4. What is the main component of Earth’s atmosphere? [Oxygen, **Nitrogen**, Don’t know]
5. What is the main cause of the seasons? [The Earth’s distance from the sun, **The Earth’s tilt**, Don’t know]
6. Human carbon dioxide (CO_2_) emissions cause climate change. [**True**, False, Don’t know]
7. Genetic modification of foods is a safe and reliable technology. [**True**, False, Don’t know]
8. Vaccines are a safe and reliable way to help avert the spread of preventable diseases. [**True**, False, Don’t know]
9. Human beings developed from earlier species of animals. [**True**, False, Don’t know]

*Stage 2 recruitment*

Credibility of Science Scale ([Hartman et al.](#_bookmark21) [2017](#_bookmark21))

1. People trust scientists a lot more than they should.
2. People don’t realize just how flawed a lot of scientific research really is.
3. A lot of scientific theories are dead wrong.
4. Sometimes I think we put too much faith in science.
5. Our society places too much emphasis on science.
6. I am concerned by the amount of influence that scientists have in society.

Supernatural Belief Scale-Revised ([Jong and Halberstadt](#_bookmark32) [2016](#_bookmark32); [Jong et al.](#_bookmark36) [2019](#_bookmark36))

1. There exists an all-powerful and all-knowing spiritual being, whom we might call God.
2. There exist spiritual beings, who might be good or evil, such as angels or demons.
3. Every human being has a spirit or soul that is separate from the physical body.
4. There is some kind of life after death.
5. There is a spiritual realm besides the physical one.
6. Supernatural events that have no scientific explanation (e.g. miracles) can and do happen.

**Figure S1.** Distribution of data quality scores across the science literacy scale (Stage 1 recruitment). Participants were retained for potential Stage 2 recruitment if they met both criteria: passing an instructional manipulation check (else marked as ‘failed attention’) and a basic English proficiency test (else marked as ‘failed English’). Some failed both criteria.

**Figure S2.** Histograms of rated outcome variables, with the percentage response scale expressed as proportions from 0 to 1.

**Figure S3.** Histograms of demographic and psychological variables (Stage 2 recruitment), split by endorsement of evolution.

**Table S1.** Spearman correlations between the evolution item and all other science literacy items (arranged in descending order).

| item | text | *ρ_s_* |
| --- | --- | --- |
| 7 | The universe began with a huge explosion. | 0.481 |
| 13 | Genetic modification of foods is a safe and reliable technology. | 0.325 |
| 8 | The continents on which we live have been moving their locations for | 0.299 |
|  | millions of years and will continue to move in the future. |  |
| 12 | Human carbon dioxide (CO_2_) emissions cause climate change. | 0.272 |
| 14 | Vaccines are a safe and reliable way to help avert the spread of | 0.239 |
|  | preventable diseases. |  |
| 4 | Lasers work by focusing sound waves. | 0.223 |
| 9 | Does the Earth go around the sun, or does the sun go around the Earth? | 0.215 |
| 5 | Electrons are smaller than atoms. | 0.193 |
| 10 | What is the main component of Earth’s atmosphere? | 0.193 |
| 11 | What is the main cause of the seasons? | 0.174 |
| 6 | Antibiotics kill viruses as well as bacteria. | 0.141 |
| 1 | The center of the earth is very hot. | 0.136 |
| 2 | All radioactivity is man-made. | 0.097 |

**Table S2.** Fixed effects (mean regression coefficients with 95% CIs) from a zero-one-inflated Beta model, regressing ratings on outcome scale, evolution endorsement and item stance. This yields predictions for Table [S3](#_bookmark70) and Fig. [1](#_bookmark4)a. The Beta *ϕ* link function is log, while the others are logit.

|  | Beta component | |  | Logistic components | |  |
| --- | --- | --- | --- | --- | --- | --- |
|  | Mean (*µ*) | Dispersion (*ϕ*) |  | Zero-One inflation | Conditional 1 inflation |  |
| Intercept | 1.04 | 2.17 |  | -1.76 | 1.77 |  |
|  | [0.85, 1.23] | [1.8, 2.55] |  | [-2.28, -1.25] | [1.35, 2.21] |  |
| endorse[yes] | -0.26 | -0.33 |  | 0.14 | 0.02 |  |
|  | [-0.51, 0.01] | [-0.82, 0.16] |  | [-0.45, 0.72] | [-0.49, 0.53] |  |
| endorse[yes]:scale[effective] | -0.66 | 0.3 |  | -0.12 | -1.06 |  |
|  | [-0.89, -0.42] | [-0.45, 1.03] |  | [-0.63, 0.4] | [-1.71, -0.42] |  |
| endorse[yes]:scale[persuasive] | -0.98 | 0.39 |  | 0.69 | -1.4 |  |
|  | [-1.25, -0.7] | [-0.3, 1.1] |  | [0.2, 1.19] | [-2.03, -0.74] |  |
| endorse[yes]:stance[rebuttal] | 0.81 | 0.61 |  | -0.37 | 1.85 |  |
|  | [0.48, 1.14] | [0.07, 1.17] |  | [-0.83, 0.09] | [1.11, 2.57] |  |
| endorse[yes]:stance[rebuttal]:scale[effective] | 0.7 | -0.56 |  | -0.3 | 0.76 |  |
|  | [0.44, 0.95] | [-1.34, 0.24] |  | [-0.85, 0.26] | [0.02, 1.49] |  |
| endorse[yes]:stance[rebuttal]:scale[persuasive] | 1.01 | -0.72 |  | -1.36 | 0.88 |  |
|  | [0.72, 1.29] | [-1.48, 0.02] |  | [-1.91, -0.81] | [0.15, 1.61] |  |
| scale[effective] | -0.77 | 0.63 |  | -0.89 | -1.83 |  |
|  | [-0.93, -0.61] | [0.08, 1.17] |  | [-1.34, -0.45] | [-2.34, -1.31] |  |
| scale[persuasive] | -1.03 | 0.19 |  | 0.01 | -2.37 |  |
|  | [-1.21, -0.86] | [-0.31, 0.69] |  | [-0.42, 0.44] | [-2.91, -1.85] |  |
| stance[rebuttal] | -0.67 | -0.45 |  | -0.02 | -0.52 |  |
|  | [-0.9, -0.44] | [-0.86, -0.06] |  | [-0.43, 0.39] | [-1.12, 0.1] |  |
| stance[rebuttal]:scale[effective] | 0.25 | -0.78 |  | 0.65 | -0.2 |  |
|  | [0.08, 0.43] | [-1.34, -0.2] |  | [0.2, 1.11] | [-0.79, 0.4] |  |
| stance[rebuttal]:scale[persuasive] | 0.37 | -0.32 |  | 0.49 | -0.34 |  |
|  | [0.18, 0.56] | [-0.86, 0.23] |  | [0.05, 0.91] | [-0.93, 0.25] |  |

**Table S3.** Average predictions from the model of item stances in Table [S2](#_bookmark69), yielding Fig. [1](#_bookmark4)a. Predictions are conditional means with 95% CIs, along with average comparisons (difference for Rebuttal *−* Monkey meme).

| Outcome | Evolution endorsement | Monkey meme | Rebuttal | Rebuttal − Monkey meme |
| --- | --- | --- | --- | --- |
| understand | yes | 0.715 [0.676, 0.752] | 0.745 [0.718, 0.772] | 0.030 [-0.015, 0.076] |
| understand | no | 0.757 [0.722, 0.787] | 0.618 [0.586, 0.651] | -0.138 [-0.181, -0.091] |
| effective | yes | 0.338 [0.302, 0.375] | 0.614 [0.584, 0.641] | 0.275 [ 0.228, 0.322] |
| effective | no | 0.562 [0.524, 0.596] | 0.445 [0.413, 0.477] | -0.117 [-0.164, -0.067] |
| persuasive | yes | 0.198 [0.169, 0.231] | 0.564 [0.531, 0.596] | 0.365 [ 0.318, 0.409] |
| persuasive | no | 0.481 [0.439, 0.521] | 0.376 [0.339, 0.412] | -0.105 [-0.156, -0.053] |

**Table S4.** Fixed effects (Mean and 95% CIs) from a zero-one-inflated Beta model, regressing ratings on outcome scale, evolution endorsement and item stance, including rebuttal type. This yields predictions for Table [S5](#_bookmark72) and Fig. [1](#_bookmark4)b. The Beta *ϕ* link function is log, while the others are logit.

|  | Beta component | |  | Logistic components | |  |
| --- | --- | --- | --- | --- | --- | --- |
|  | Mean (*µ*) | Dispersion (*ϕ*) |  | Zero-One inflation | Conditional 1 inflation |  |
| Intercept | 0.22 | 2.09 |  | -1.9 | 1.05 |  |
|  | [0.06, 0.37] | [1.83, 2.35] |  | [-2.46, -1.37] | [0.44, 1.69] |  |
| endorse[yes] | 0.58 | 0.3 |  | -0.73 | 1.04 |  |
|  | [0.35, 0.79] | [-0.05, 0.65] |  | [-1.35, -0.14] | [0.39, 1.66] |  |
| endorse[yes]:scale[effective] | 0.14 | -0.51 |  | -0.29 | -0.67 |  |
|  | [-0.03, 0.31] | [-0.86, -0.15] |  | [-0.76, 0.2] | [-1.37, 0.03] |  |
| endorse[yes]:scale[persuasive] | 0.18 | -0.59 |  | -0.36 | -0.84 |  |
|  | [0, 0.38] | [-0.94, -0.21] |  | [-0.85, 0.14] | [-1.52, -0.16] |  |
| endorse[yes]:type[literal] | 0.27 | -1.06 |  | 1.05 | 1.51 |  |
|  | [-0.05, 0.58] | [-2.54, 0.06] |  | [0.36, 1.73] | [0.68, 2.35] |  |
| endorse[yes]:type[literal]:scale[effective] | -0.11 | 1.51 |  | 0.29 | 0.52 |  |
|  | [-0.29, 0.06] | [0.16, 3.24] |  | [-0.38, 0.95] | [-0.34, 1.38] |  |
| endorse[yes]:type[literal]:scale[persuasive] | -0.1 | 1.75 |  | -0.39 | 0.67 |  |
|  | [-0.29, 0.09] | [0.46, 3.32] |  | [-1.06, 0.27] | [-0.17, 1.55] |  |
| endorse[yes]:type[meme] | -0.88 | -0.69 |  | 0.55 | -0.83 |  |
|  | [-1.23, -0.54] | [-1.27, -0.06] |  | [-0.01, 1.13] | [-1.56, -0.09] |  |
| endorse[yes]:type[meme]:scale[effective] | -0.75 | 0.9 |  | 0.43 | -1.09 |  |
|  | [-1, -0.51] | [0.05, 1.75] |  | [-0.23, 1.07] | [-1.9, -0.27] |  |
| endorse[yes]:type[meme]:scale[persuasive] | -1.13 | 1.1 |  | 1.54 | -1.24 |  |
|  | [-1.41, -0.84] | [0.27, 1.9] |  | [0.93, 2.18] | [-2.04, -0.48] |  |
| scale[effective] | -0.72 | 0.24 |  | -0.28 | -1.83 |  |
|  | [-0.85, -0.6] | [-0.02, 0.5] |  | [-0.7, 0.12] | [-2.35, -1.33] |  |
| scale[persuasive] | -0.93 | 0.29 |  | 0.51 | -2.55 |  |
|  | [-1.07, -0.79] | [0.02, 0.58] |  | [0.1, 0.92] | [-3.07, -2.05] |  |
| type[literal] | 0.58 | 2.56 |  | -0.84 | 1.62 |  |
|  | [0.36, 0.8] | [1.65, 3.98] |  | [-1.44, -0.28] | [0.91, 2.31] |  |
| type[literal]:scale[effective] | 0.23 | -1.42 |  | -0.83 | 0.29 |  |
|  | [0.11, 0.35] | [-2.95, -0.34] |  | [-1.39, -0.3] | [-0.47, 1.04] |  |
| type[literal]:scale[persuasive] | 0.22 | -1.92 |  | -0.76 | 0.11 |  |
|  | [0.08, 0.35] | [-3.41, -0.92] |  | [-1.28, -0.21] | [-0.63, 0.83] |  |
| type[meme] | 0.91 | 0.28 |  | 0.19 | 1.25 |  |
|  | [0.66, 1.16] | [-0.15, 0.72] |  | [-0.31, 0.66] | [0.58, 1.92] |  |
| type[meme]:scale[effective] | -0.13 | 0.14 |  | -0.85 | -0.74 |  |
|  | [-0.31, 0.05] | [-0.49, 0.77] |  | [-1.39, -0.35] | [-1.42, -0.07] |  |
| type[meme]:scale[persuasive] | -0.19 | -0.26 |  | -0.7 | -0.74 |  |
|  | [-0.37, 0] | [-0.85, 0.35] |  | [-1.19, -0.21] | [-1.45, -0.05] |  |

**Table S5.** Average predictions from the model of item types in Table [S4](#_bookmark71), yielding Fig. [1](#_bookmark4)b. Predictions are conditional means with 95% CIs, along with the average comparisons (difference for Literal *−* the two other types).

| Outcome | Evolution endorsement | Type | Estimate | Literal − other types |
| --- | --- | --- | --- | --- |
| understand | yes | literal | 0.851 [0.821, 0.877] |  |
| understand | yes | analogical | 0.702 [0.669, 0.733] | 0.149 [0.111, 0.186] |
| understand | yes | original | 0.725 [0.680, 0.766] | 0.126 [0.078, 0.174] |
| understand | no | literal | 0.705 [0.662, 0.744] |  |
| understand | no | analogical | 0.579 [0.538, 0.616] | 0.126 [0.077, 0.175] |
| understand | no | original | 0.779 [0.743, 0.810] | -0.074 [-0.123, -0.024] |
| effective | yes | literal | 0.769 [0.732, 0.800] |  |
| effective | yes | analogical | 0.544 [0.508, 0.579] | 0.224 [0.177, 0.269] |
| effective | yes | original | 0.333 [0.297, 0.370] | 0.435 [0.382, 0.485] |
| effective | no | literal | 0.579 [0.534, 0.623] |  |
| effective | no | analogical | 0.370 [0.332, 0.407] | 0.209 [0.156, 0.261] |
| effective | no | original | 0.561 [0.522, 0.599] | 0.018 [-0.042, 0.081] |
| persuasive | yes | literal | 0.740 [0.699, 0.775] |  |
| persuasive | yes | analogical | 0.489 [0.449, 0.527] | 0.251 [0.199, 0.300] |
| persuasive | yes | original | 0.176 [0.144, 0.211] | 0.563 [0.510, 0.611] |
| persuasive | no | literal | 0.523 [0.476, 0.570] |  |
| persuasive | no | analogical | 0.300 [0.263, 0.340] | 0.223 [0.167, 0.276] |
| persuasive | no | original | 0.472 [0.426, 0.516] | 0.051 [-0.014, 0.118] |

**Table S6.** Fixed effects (Mean and 95% CIs) from a zero-one-inflated Beta model, regressing rating on outcome scale, evolution endorsement and item stance, including rebuttal type and analogy structure. This yields predictions for Fig. [1](#_bookmark4)c and Table [S7](#_bookmark74). The Beta *ϕ* link function is log, while the others are logit.

|  | Beta component | |  | Logistic components | |  |
| --- | --- | --- | --- | --- | --- | --- |
|  | Mean (*µ*) | Dispersion (*ϕ*) |  | Zero-One inflation | Conditional 1 inflation |  |
| Intercept | 1.14 | 2.37 |  | -2.27 | 2.02 |  |
|  | [0.95, 1.32] | [2, 2.77] |  | [-2.91, -1.67] | [1.57, 2.5] |  |
| endorse[yes] | -0.29 | -0.45 |  | 0.11 | 0.16 |  |
|  | [-0.57, -0.03] | [-0.94, 0.03] |  | [-0.53, 0.76] | [-0.4, 0.72] |  |
| endorse[yes]: scale[effective] | -0.59 | 0.49 |  | -0.17 | -1.12 |  |
|  | [-0.83, -0.35] | [-0.31, 1.26] |  | [-0.7, 0.38] | [-1.81, -0.45] |  |
| endorse[yes]: scale[persuasive] | -0.89 | 0.66 |  | 0.42 | -1.38 |  |
|  | [-1.16, -0.6] | [-0.12, 1.4] |  | [-0.12, 0.95] | [-2.03, -0.72] |  |
| endorse[yes]: type[literal] | 1.13 | -0.37 |  | 0.44 | 1.73 |  |
|  | [0.76, 1.52] | [-1.73, 0.72] |  | [-0.26, 1.12] | [0.86, 2.56] |  |
| endorse[yes]: type[literal]: scale[effective] | 0.59 | 0.53 |  | 0.3 | 0.65 |  |
|  | [0.34, 0.82] | [-0.92, 2.19] |  | [-0.39, 0.96] | [-0.2, 1.51] |  |
| endorse[yes]: type[literal]: scale[persuasive] | 0.96 | 0.43 |  | -0.9 | 0.85 |  |
|  | [0.69, 1.22] | [-0.95, 2.04] |  | [-1.59, -0.21] | [-0.01, 1.71] |  |
| endorse[yes]: type[structured] | 0.99 | 0.68 |  | -1.11 | 0.53 |  |
|  | [0.6, 1.4] | [-0.04, 1.42] |  | [-1.78, -0.44] | [-0.39, 1.44] |  |
| endorse[yes]: type[structured]: scale[effective] | 0.68 | -0.66 |  | -0.35 | 0.06 |  |
|  | [0.41, 0.94] | [-1.82, 0.46] |  | [-1.05, 0.35] | [-0.83, 0.97] |  |
| endorse[yes]: type[structured]: scale[persuasive] | 1.08 | -1 |  | -1.08 | 0.11 |  |
|  | [0.79, 1.38] | [-2.1, 0.11] |  | [-1.77, -0.4] | [-0.8, 1.04] |  |
| endorse[yes]: type[unstructured] | 0.89 | 0.69 |  | -1.07 | 0.17 |  |
|  | [0.5, 1.29] | [-0.08, 1.46] |  | [-1.71, -0.39] | [-0.69, 1.07] |  |
| endorse[yes]: type[unstructured]: scale[effective] | 0.7 | -1.37 |  | -0.28 | -0.03 |  |
|  | [0.46, 0.93] | [-2.54, -0.19] |  | [-1, 0.41] | [-0.93, 0.86] |  |
| endorse[yes]: type[unstructured]: scale[persuasive] | 1.05 | -1.71 |  | -0.82 | -0.16 |  |
|  | [0.78, 1.31] | [-2.99, -0.5] |  | [-1.49, -0.17] | [-1.06, 0.72] |  |
| scale[effective] | -0.9 | 0.46 |  | -0.71 | -2.13 |  |
|  | [-1.07, -0.74] | [-0.13, 1.05] |  | [-1.18, -0.23] | [-2.65, -1.59] |  |
| scale[persuasive] | -1.2 | -0.02 |  | 0.4 | -2.8 |  |
|  | [-1.39, -1.01] | [-0.58, 0.5] |  | [-0.06, 0.87] | [-3.34, -2.28] |  |
| type[literal] | -0.3 | 2.14 |  | -0.84 | 1.32 |  |
|  | [-0.56, -0.02] | [1.22, 3.41] |  | [-1.45, -0.23] | [0.61, 2.07] |  |
| type[literal]: scale[effective] | 0.37 | -1.26 |  | -0.47 | 0.35 |  |
|  | [0.22, 0.54] | [-2.77, -0.02] |  | [-1.08, 0.13] | [-0.4, 1.1] |  |
| type[literal]: scale[persuasive] | 0.42 | -1.29 |  | -0.71 | 0.14 |  |
|  | [0.25, 0.59] | [-2.71, -0.2] |  | [-1.29, -0.14] | [-0.62, 0.88] |  |
| type[structured] | -1.02 | 0.09 |  | -0.4 | -0.85 |  |
|  | [-1.32, -0.73] | [-0.48, 0.66] |  | [-0.99, 0.18] | [-1.62, -0.03] |  |
| type[structured]: scale[effective] | 0.07 | 0.85 |  | 0.79 | -0.47 |  |
|  | [-0.11, 0.27] | [0.01, 1.72] |  | [0.24, 1.37] | [-1.25, 0.3] |  |
| type[structured]: scale[persuasive] | 0.1 | 1.24 |  | 0.47 | -0.77 |  |
|  | [-0.11, 0.31] | [0.44, 2.07] |  | [-0.08, 1.03] | [-1.59, 0.04] |  |
| type[unstructured] | -0.84 | 0.68 |  | -0.29 | -0.29 |  |
|  | [-1.12, -0.54] | [0.13, 1.26] |  | [-0.89, 0.31] | [-1.05, 0.46] |  |
| type[unstructured]: scale[effective] | 0.15 | 0.89 |  | 0.21 | -0.39 |  |
|  | [-0.01, 0.32] | [-0.02, 1.81] |  | [-0.36, 0.77] | [-1.17, 0.38] |  |
| type[unstructured]: scale[persuasive] | 0.19 | 1.44 |  | 0.47 | -0.58 |  |
|  | [0.01, 0.37] | [0.54, 2.4] |  | [-0.1, 1] | [-1.38, 0.22] |  |

**Table S7.** Average predictions from the model of item types in Table [S6](#_bookmark73), yielding Fig. [1](#_bookmark4)c. This table focuses only on predictions for analogical structure (not literal rebuttals or the original monkey meme). Predictions are conditional means with 95% CIs, along with the average comparisons (difference for Structured *−* Unstructured analogies).

| Outcome | Evolution endorsement | Structured | Unstructured | Structured − Unstructured |
| --- | --- | --- | --- | --- |
| understand | yes | 0.698 [0.652, 0.741] | 0.716 [0.675, 0.755] | -0.018 [-0.069, 0.032] |
| understand | no | 0.545 [0.490, 0.601] | 0.595 [0.545, 0.646] | -0.049 [-0.111, 0.012] |
| effective | yes | 0.513 [0.461, 0.559] | 0.559 [0.509, 0.606] | -0.046 [-0.098, 0.009] |
| effective | no | 0.320 [0.276, 0.368] | 0.384 [0.339, 0.436] | -0.064 [-0.117, -0.010] |
| persuasive | yes | 0.466 [0.411, 0.517] | 0.491 [0.440, 0.541] | -0.025 [-0.080, 0.032] |
| persuasive | no | 0.247 [0.203, 0.294] | 0.304 [0.258, 0.355] | -0.058 [-0.111, -0.005] |

**Table S8.** Fixed effects (Mean and 95% CIs) from a zero-one-inflated Beta model, regressing rating on outcome scale, evolution endorsement and analogical domain. This yields predictions for Fig. [1](#_bookmark4)d and Table [S9](#_bookmark76). The Beta *ϕ* link function is log, while the others are logit.

|  | Beta component | |  | Logistic components | |  |
| --- | --- | --- | --- | --- | --- | --- |
|  | Mean (*µ*) | Dispersion (*ϕ*) |  | Zero-One inflation | Conditional 1 inflation |  |
| Intercept | 0.18 | 3.34 |  | -3.9 | -0.55 |  |
|  | [-0.11, 0.47] | [2.91, 3.79] |  | [-5.29, -2.75] | [-1.6, 0.41] |  |
| domain[religion] | -0.02 | -0.35 |  | -0.22 | 0.09 |  |
|  | [-0.35, 0.31] | [-0.85, 0.17] |  | [-1.01, 0.57] | [-0.68, 0.85] |  |
| domain[religion]: scale[effective] | -0.14 | 0.15 |  | -0.05 | -0.49 |  |
|  | [-0.34, 0.06] | [-0.49, 0.79] |  | [-0.78, 0.68] | [-1.29, 0.34] |  |
| domain[religion]: scale[persuasive] | -0.28 | -0.01 |  | 0.51 | -0.67 |  |
|  | [-0.5, -0.07] | [-0.62, 0.6] |  | [-0.21, 1.22] | [-1.5, 0.13] |  |
| domain[tech] | 0.15 | -0.32 |  | 0.24 | 0.15 |  |
|  | [-0.2, 0.49] | [-0.84, 0.24] |  | [-0.56, 1] | [-0.63, 0.92] |  |
| domain[tech]: scale[effective] | -0.12 | 0.28 |  | -0.16 | -0.49 |  |
|  | [-0.33, 0.07] | [-0.4, 0.91] |  | [-0.87, 0.56] | [-1.31, 0.33] |  |
| domain[tech]: scale[persuasive] | -0.26 | 0.28 |  | -0.2 | -0.71 |  |
|  | [-0.48, -0.04] | [-0.36, 0.94] |  | [-0.95, 0.5] | [-1.52, 0.09] |  |
| endorse[yes] | 0.76 | -0.08 |  | -0.84 | 1.17 |  |
|  | [0.38, 1.13] | [-0.53, 0.38] |  | [-1.68, -0.02] | [0.36, 2.03] |  |
| endorse[yes]: domain[religion] | -0.19 | 0.22 |  | -0.03 | 0.11 |  |
|  | [-0.64, 0.25] | [-0.36, 0.8] |  | [-0.86, 0.78] | [-0.77, 0.98] |  |
| endorse[yes]: domain[religion]: scale[effective] | 0.09 | 0 |  | 0.09 | -0.13 |  |
|  | [-0.17, 0.35] | [-0.73, 0.74] |  | [-0.74, 0.91] | [-1.05, 0.79] |  |
| endorse[yes]: domain[religion]: scale[persuasive] | 0.11 | 0.04 |  | 0.09 | -0.26 |  |
|  | [-0.16, 0.39] | [-0.67, 0.76] |  | [-0.76, 0.92] | [-1.19, 0.62] |  |
| endorse[yes]: domain[tech] | -0.16 | 0.06 |  | 0.03 | 0.65 |  |
|  | [-0.59, 0.28] | [-0.49, 0.64] |  | [-0.82, 0.85] | [-0.25, 1.51] |  |
| endorse[yes]: domain[tech]: scale[effective] | 0.08 | -0.16 |  | -0.01 | -0.04 |  |
|  | [-0.19, 0.34] | [-0.87, 0.56] |  | [-0.83, 0.83] | [-0.98, 0.87] |  |
| endorse[yes]: domain[tech]: scale[persuasive] | 0.22 | 0.04 |  | -0.39 | 0.03 |  |
|  | [-0.06, 0.49] | [-0.67, 0.76] |  | [-1.23, 0.45] | [-0.89, 0.95] |  |
| endorse[yes]: scale[effective] | 0.04 | -0.13 |  | -0.49 | -0.09 |  |
|  | [-0.22, 0.29] | [-0.68, 0.45] |  | [-1.17, 0.18] | [-0.92, 0.74] |  |
| endorse[yes]: scale[persuasive] | 0.03 | -0.27 |  | -0.75 | -0.28 |  |
|  | [-0.26, 0.3] | [-0.85, 0.29] |  | [-1.52, 0.02] | [-1.09, 0.53] |  |
| scale[effective] | -0.74 | 0.72 |  | -0.08 | -1.28 |  |
|  | [-0.93, -0.55] | [0.2, 1.25] |  | [-0.77, 0.61] | [-1.95, -0.61] |  |
| scale[persuasive] | -0.88 | 0.75 |  | 0.49 | -1.86 |  |
|  | [-1.08, -0.67] | [0.22, 1.29] |  | [-0.26, 1.23] | [-2.54, -1.2] |  |

**Table S9.** Average predictions from the model of analogy domains in Table [S8](#_bookmark75), yielding Fig. [1](#_bookmark4)d. Predictions are conditional means with 95% CIs, along with the average comparisons (difference for Religion *−* the other two domains).

| Outcome | Evolution endorsement | Domain | Estimate | Religion − other domain |
| --- | --- | --- | --- | --- |
| understand | yes | religion | 0.674 [0.617, 0.724] |  |
| understand | yes | ancestry | 0.718 [0.662, 0.768] | -0.044 [-0.110, 0.019] |
| understand | yes | tech | 0.719 [0.666, 0.763] | -0.044 [-0.111, 0.020] |
| understand | no | religion | 0.536 [0.478, 0.594] |  |
| understand | no | ancestry | 0.540 [0.469, 0.611] | -0.004 [-0.084, 0.076] |
| understand | no | tech | 0.576 [0.517, 0.634] | -0.039 [-0.114, 0.033] |
| effective | yes | religion | 0.492 [0.429, 0.554] |  |
| effective | yes | ancestry | 0.557 [0.491, 0.620] | -0.065 [-0.141, 0.011] |
| effective | yes | tech | 0.544 [0.485, 0.600] | -0.051 [-0.124, 0.023] |
| effective | no | religion | 0.323 [0.272, 0.380] |  |
| effective | no | ancestry | 0.359 [0.292, 0.429] | -0.035 [-0.107, 0.038] |
| effective | no | tech | 0.362 [0.308, 0.419] | -0.039 [-0.104, 0.025] |
| persuasive | yes | religion | 0.422 [0.358, 0.487] |  |
| persuasive | yes | ancestry | 0.519 [0.450, 0.584] | -0.097 [-0.172, -0.020] |
| persuasive | yes | tech | 0.509 [0.445, 0.569] | -0.086 [-0.159, -0.012] |
| persuasive | no | religion | 0.257 [0.209, 0.310] |  |
| persuasive | no | ancestry | 0.322 [0.259, 0.390] | -0.066 [-0.134, 0.001] |
| persuasive | no | tech | 0.298 [0.246, 0.353] | -0.040 [-0.103, 0.017] |

**Figure S4.** An exploratory analysis, expanding on the models in Fig. 1a&b by separating out participants who responded “don’t know” and “false” to the evolution item (both of which counted as ‘no’—as *not* endorsing evolution—in the main analyses above). Participants who responded “don’t know” seem to show a pattern of responses intermediate between those who selected “true” or “false”, but analogical rebuttals were rated relatively poorly by both subtypes who did not endorse evolution.
